# Normocalcemic primary hyperparathyroidism

**DOI:** 10.20945/2359-3997000000556

**Published:** 2022-11-10

**Authors:** Natalie E. Cusano, Filomena Cetani

**Affiliations:** 1 Lenox Hill Hospital Department of Medicine Division of Endocrinology New York NY USA Division of Endocrinology, Department of Medicine, Lenox Hill Hospital, New York, NY, USA; 2 University Hospital of Pisa Endocrine Unit Pisa Italy Endocrine Unit, University Hospital of Pisa, Pisa, Italy

**Keywords:** Hyperparathyroidism, osteoporosis, nephrolithiasis, parathyroid surgery, calcium, parathyroid hormone

## Abstract

Normocalcemic primary hyperparathyroidism (PHPT) is a newer phenotype of PHPT defined by elevated PTH concentrations in the setting of normal serum calcium levels. It is increasingly being diagnosed in the setting of evaluation for nephrolithiasis or metabolic bone diseases. It is important to demonstrate that PTH values remain consistently elevated and to measure ionized calcium levels to make the diagnosis. A diagnosis of normocalcemic disease is one of exclusion of secondary forms of hyperparathyroidism, including vitamin D deficiency, renal failure, medications, malabsorption, and hypercalciuria. Lack of rigorous diagnostic criteria and selection bias of the studied populations may explain the different rates of bone and renal complications. The natural history still remains unknown. Caution should be used in recommending surgery, unless clearly indicated. Here we will review the diagnostic features, epidemiology, clinical presentation, natural history, medical and surgical management of normocalcemic PHPT.

## INTRODUCTION

Primary hyperparathyroidism (PHPT) is a common endocrine disorder. In the developed world is now usually an incidental diagnosis made when asymptomatic hypercalcemia is detected on routine blood tests. Symptomatic disease, defined by kidney stones and fractures and remembered by the mnemonic “bones, stones, abdominal groans, and psychiatric overtones,” is still prevalent in some areas of the world, including South America, the Middle East, and Asia (1).

A newer phenotype of the disease is being described, primarily in patients presenting with nephrolithiasis or osteoporosis. Normocalcemic PHPT can be diagnosed in patients with elevated parathyroid hormone (PTH) concentrations in the setting of persistently normal serum total and ionized calcium levels. Normocalcemic PHPT was first formally recognized at the time of the Third International Workshop on the Management of Asymptomatic Primary Hyperparathyroidism in 2008 (2). Recommendations for diagnosis and management were made at the time of the Fourth International Workshop in 2013, however, there remain limited data on which to base recommendations for this phenotype (3,4). In this manuscript we will review the available literature regarding diagnostic features, epidemiology, clinical presentation, natural history, medical and surgical management of normocalcemic PHPT.

### Diagnosis

A review of the available literature regarding normocalcemic PHPT is complicated due to a lack of consistency in diagnostic criteria. Diagnostic recommendations were made at the time of the Fourth International Workshop on the Management of Asymptomatic Primary Hyperparathyroidism in 2013 (3,4) and the European Society of Endocrinology Educational Program of Parathyroid Disorders in 2021 (5). A consistent diagnosis is needed moving forward for research investigations to better guide physicians regarding management decisions.

The guidelines generally recommend that a diagnosis of normocalcemic PHPT be made in individuals with an elevated PTH concentration on multiple occasions at least three months apart, in the setting of consistently normal serum calcium (both total and ionized serum calcium). The importance of repeated measurements has been demonstrated by a lack of persistence of hyperparathyroidism in some patients diagnosed with normocalcemic disease and concern for misclassification of individuals with secondary hyperparathyroidism. In a population-based study of disease prevalence, 108 of 3450 (3.1%) men and women were noted to have an elevated PTH concentration with normal serum calcium, after exclusion of secondary causes of hyperparathyroidism. Of the 64 individuals with follow-up data eight years later, only 13 (0.6%) had persistent hyperparathyroidism (6). Patients with typical hypercalcemic PHPT can occasionally have normal serum calcium levels and should not be classified as having normocalcemic disease. Ionized calcium should be measured as part of the evaluation of a patient suspected of having normocalcemic disease, since 4-64% of patients with a diagnosis of normocalcemic PHPT can be reclassified as having traditional hypercalcemic disease with measurement of ionized calcium levels (7–9).

Normocalcemic PHPT is a diagnosis of exclusion. Secondary causes of hyperparathyroidism are common, and a rigorous exclusion of secondary causes is needed.

1. Vitamin D deficiency. Vitamin D is a common secondary cause of hyperparathyroidism, and it may take months for secondary hyperparathyroidism to resolve after treatment of vitamin D deficiency. The National Academy of Medicine (formally the Institute of Medicine) acknowledged that the literature provides no widespread agreement regarding a 25-hydroxyvitamin D level by which there is a plateau in PTH concentrations, suggesting that a range of values may be needed for different individuals (9). The 25-hydroxyvitamin D level should be at minimum 20 ng/mL as per the Fourth International Workshop guidelines (3), and at least 30 ng/mL as per the European Society of Endocrinology Expert Consensus to make a diagnosis of normocalcemic disease (5). In addition, patients with the traditional phenotype may become hypercalcemic with resolution of vitamin D deficiency.
2. Renal failure. PTH levels rise as eGFR falls. To make a diagnosis of normocalcemic PHPT, eGFR should be at least 60 mL/min to exclude stage 3-5 chronic kidney disease as a cause of hyperparathyroidism (10,11).
3. Medications. Antiresorptive medications, including bisphosphonates and denosumab, are a known cause of hyperparathyroidism by blocking calcium release from the skeleton (12–14). With zoledronic acid infusion, elevated PTH concentrations can persist for up to a year after the dose. Denosumab results in an exuberant increase in PTH concentrations peaking at around 3 months after the dose, with subsequently decline until the next dose. By shifting calcium sensing, lithium can result in hyperparathyroidism (15). The relationship between the diuretics hydrochlorothiazide and furosemide and PTH concentrations is less clear (16,17). It is recommended to exclude diuretics as a cause of hyperparathyroidism. Newer data also indicate that proton pump inhibitors (18) and SGLT-2 inhibitors (19) may also result in elevated PTH concentrations.
4. Insufficient calcium intake or malabsorption. Low calcium intake, celiac disease, and gastric bypass procedures can result in hyperparathyroidism (20,21). Patients should be advised to have sufficient calcium intake through diet and/or supplements.
5. Hypercalciuria. It has been recommended to exclude hypercalciuria, defined as a 24-hour urine calcium excretion >250 mg for women or >300 mg for men (22). The European Society of Endocrinology Expert Consensus recommends a trial of hydrochlorothiazide to determine if improving the hypercalciuria can resolve the elevation in PTH concentrations (5).
6. Disorders of phosphate metabolism. Since extracellular phosphate regulation involves change in PTH concentrations, both hypo- and hyperphosphatemia can cause hyperparathyroidism (23). The European Society of Endocrinology Expert Consensus recommends exclusion of disorders of phosphate metabolism for a diagnosis of normocalcemic PHPT.

### Epidemiology

There are few population-based studies investigating the prevalence of normocalcemic PHPT in healthy individuals since PTH concentrations are not often ordered in the absence of hypercalcemia or symptoms. As such, it is difficult to describe the epidemiology in a general healthy population. In addition, few studies have measured ionized calcium levels and there are significant differences in the literature in the exclusion of secondary causes of hyperparathyroidism, especially in the cutoffs for 25-hydroxyvitamin D and eGFR. Of note, few studies have monitored biochemical parameters over time, which can overestimate the disease prevalence. The disease appears to be relatively rare, with most studies using established criteria demonstrating a prevalence of 0.1%-0.7% (6,24–33). The epidemiologic data are summarized in Table 1.

**Table 1:** Epidemiology of normocalcemic primary hyperparathyroidism

Study	Population	Prevalence	Diagnostic criteria
Palermo *et al.* (24)	2677 community dwelling women investigated for a bone imaging study [The Osteoporosis and Ultrasound Study (OPUS), United Kingdom, France, Germany]	0.1% (Baseline) 0.0% (Follow-up)	Elevated PTH concentration and normal albumin-adjusted total serum calcium on at least two occasions; exclusion of renal failure (GFR < 60 mL/min), vitamin D deficiency (25-hydroxyvitamin D < 20 ng/mL); medication effect not excluded
Schini *et al.* (26)	6,280 men and women referred for bone density testing (United Kingdom)	0.2%	Elevated PTH concentration and normal albumin-adjusted total serum calcium on at least two occasions; exclusion of renal failure (GFR < 60 mL/min), vitamin D deficiency (25-hydroxyvitamin D < 20 ng/mL)
Wu and Anpalahan (27)	2,593 men and women from a laboratory database	0.4%	Elevated PTH concentration and normal albumin-adjusted total serum calcium and ionized calcium; exclusion of renal failure (GFR < 60 mL/min), vitamin D deficiency (25-hydroxyvitamin D < 20 ng/mL), medication effect, gastric bypass surgery
Cusano *et al.* (6)	2,364 unselected community dwelling men, age 65 years or older, investigated for fracture risk factors [The Osteoporotic Fractures in Men Study (MrOS) cohort, United States]	0.4%	Elevated PTH concentration and normal albumin-adjusted total serum calcium; exclusion of renal failure (GFR < 60 mL/min), vitamin D deficiency (25-hydroxyvitamin D < 20 ng/mL), medication effect
Vignali *et al.* (28)	685 adult men and women living in a village in Southern Italy (Italy)	0.4%	Elevated PTH concentration and normal albumin-adjusted total serum calcium; exclusion of renal failure (GFR < 60 mL/min), vitamin D deficiency (25-hydroxyvitamin D < 30 ng/mL), medication effect, overt gastrointestinal or metabolic bone diseases
Lundgren *et al.* (29)	5,202 postmenopausal women age 55 to 75 years attending a population-based mammography screening (Sweden)	0.5%	Elevated PTH concentration with normal albumin-adjusted total serum calcium and normal ionized calcium on repeat measure; none had history of malabsorption
Rosário and Calsolari (30)	676 adults without history of nephrolithiasis or fracture with planned thyroidectomy for thyroid disease (Brazil)	0.7% (eGFR > 60 mL/min, 25-hydroxyvitamin D > 30 ng/mL) 1.8% (eGFR > 40 mL/min, 25-hydroxyvitamin D > 30 ng/mL) 4.4% (eGFR > 60 mL/min, 25-hydroxyvitamin D > 20 ng/mL) 6.8% (eGFR >40 mL/min, 25-hydroxyvitamin D > 40 ng/mL)	Elevated PTH concentration and normal albumin-adjusted total serum calcium; exclusion of renal failure (GFR < 40 mL/min), vitamin D deficiency (25-hydroxyvitamin D < 20 ng/mL), medication effect, malabsorption (by history and measurement of tissue transglutaminase IgA)
Kontogeorgos *et al.* (31)	608 men and women, age 25-64 years, investigated for cardiovascular disease [World Health Organization MONitoring of trends and determinants for Cardiovascular disease project (WHO MONICA)]	2.0% (Baseline) 0.2% (Follow-up)	Elevated PTH concentration and normal total serum calcium; exclusion of renal failure (eGFR < 60 mL/min), vitamin D deficiency (25-hydroxyvitamin D < 20 ng/mL); medication effect not excluded
Cusano *et al.* (6)	3,450 community dwelling men and women, age 18 to 65 years, investigated for cardiovascular disease (Dallas Heart Study cohort, United States)	3.1% (Baseline) 0.6% (Follow-up)	Elevated PTH concentration and normal albumin-adjusted total serum calcium; exclusion of renal failure (eGFR < 60 mL/min), vitamin D deficiency (25-hydroxyvitamin D < 20 ng/mL), medication effect
Berger *et al.* (32)	1,872 community dwelling men and women, age 35 and older investigated for fracture risk factors (Canadian Multicentre Osteoporosis Study, Canada)	3.3%	Elevated PTH concentration and normal albumin-adjusted total serum calcium; exclusion of renal failure (eGFR < 60 mL/min), vitamin D deficiency (25-hydroxyvitamin D < 20 ng/mL); medication effect not excluded and majority of patients on diuretic and/or antiresorptive therapy
García-Martín *et al.* (33)	100 healthy, unselected postmenopausal women (Spain)	6% (Baseline) 6% (Follow-up)	Elevated PTH concentration and normal albumin-adjusted total serum calcium; exclusion of renal failure (creatinine clearance < 70 mL/min), vitamin D deficiency (25-hydroxyvitamin D < 30 ng/mL)
Marques *et al.* (25)	156 women referred for osteoporosis screening (Brazil)	8.9%	Elevated PTH concentration and normal albumin-adjusted total serum calcium; exclusion of renal failure (eGFR < 40 mL/min), vitamin D deficiency (25-hydroxyvitamin D < 30 ng/mL), history of metabolic bone disease, liver disease, malabsorption syndromes, medication effect

### Clinical presentation

Traditional hypercalcemic PHPT has now become a disease that presents primarily asymptomatically, although kidney stones or vertebral fractures are more common if screening procedures are performed to evaluate for these complications as recommended per the guidelines. Cohorts of patients with normocalcemic disease, however, typically present symptomatically. This is most likely due to selection bias, where patients with normal serum calcium are only screened for parathyroid disease in the setting of fracture or nephrolithiasis. Few population-based, unselected cohorts have been described. In these individuals identified through biochemical testing, there do not appear to be significant differences in clinical indices, bone turnover markers, or bone mass as compared to euparathyroid patients or patients with secondary hyperparathyroidism. The clinical features are summarized in Table 2 (6,25,27,30,33–41).

**Table 2:** Clinical summary of skeletal and renal complications in cohorts of patients with normocalcemic primary hyperparathyroidism

Study	Cohort Size	Age (years)	Female (%)	Osteoporosis	Nephrolithiasis	Comments
Symptomatic cohorts						
Lowe *et al.* (37)	37	58 ± 2	95	57% 11% with fragility fracture	14%	Ionized calcium not available for all
Maruani *et al.* (38)	34	53 ± 14	68	Radiographic bone demineralization in 18% (bone density not performed)	35%	
Marques *et al.* (25)	14	61 ± 15	100**	36% 21% with fragility fracture	21%	
Tordjman *et al.* (39)	32	61 ± 11	84	36%	9%	
Amaral *et al.* (40)	33	64 ± 14	79	15%* *Only fracture history available	18%	Ionized calcium not measured
Cakir *et al.* (41)	18	50 ± 2	47	47%	11%	Ionized calcium not measured
Wade *et al.* (34)	8	60	63	25% 13% with fragility fracture	25%	Surgical cohort
Šiprová *et al.* (35)	137	61	81	42^%^ described as having “reduced bone density”	4%	
Palermo *et al.* (36)	47	64 ± 9	91	28%**Only vertebral fracture history available	13%	
Wu and Anpalahan (27)	10	61.5 (49-69.8)	100%	30%	50%	
**Asymptomatic cohort**						
García-Martín *et al.* (33)	6	56 ± 3	100**	0%	0%	Ionized calcium not measured Population-based cohort
Rosário and Calsolari (30)	5	53	80%	0%	0%	Patients undergoing planned thyroid surgery 80% of patients had confirmed parathyroid pathology

Mean ± SD

** Study design

While severe traditional PHPT with significant elevations in serum calcium can result in manifestations involving multiple organ systems, studies investigating nonclassical manifestations of the mild, asymptomatic form of hypercalcemic PHPT have been mixed regarding possible complications such as hypertension, left ventricular hypertrophy, glucose intolerance, and quality of life. Nonclassical manifestations have also been studied in patients with the normocalcemic phenotype. Studies of glucose metabolism have not demonstrated any differences in hemoglobin A1c or insulin sensitivity in men and women with normocalcemic PHPT versus controls, however a few studies have shown a small but statistically significant increase in mean fasting glucose values (42–46). Studies using echocardiography or various noninvasive measures of arterial compliance as surrogate markers for vascular risk found no differences in individuals with normocalcemic disease (47,48). While one study showed an increase in coronary artery calcium score in patients with normocalcemic PHPT (49), another found no difference (50). One study demonstrated small but significant reductions in quality of life in individuals with normocalcemic disease (51).

### Natural history

There are few investigations into the natural history of normocalcemic PHPT, with most data from small cohorts showing that many patients may never develop hypercalcemia (31,33,35,37,39). In one study of individuals with normocalcemic PHPT, none of the 20 patients developed hypercalcemia over a median of four years of monitoring (39). Another study demonstrated that 7 of 37 (19%) of patients developed hypercalcemia over a median of three years (37). During this monitoring period, 41% of patients showed evidence of progressive disease, including hypercalcemia, new hypercalciuria, kidney stone, and/or fracture. In a symptomatic cohort of 187 patients with normocalcemic PHPT, 36 patients (19%) became hypercalcemic (35). The majority (67%) of these patients transitioned to hypercalcemia within the first two years of follow-up, however, a small cohort (6%) became hypercalcemic after four years. In the Dallas Heart Study cohort, only one of the initial 64 patients with a diagnosis of normocalcemic PHPT as defined developed hypercalcemia on subsequent measurement eight years later (6). In a small cohort of 6 asymptomatic postmenopausal women with normocalcemic disease, none developed evidence of hypercalcemia, nephrolithiasis, or fracture after one year (33).

### Management

#### Medical therapy

Two studies have evaluated the impact of pharmacologic treatment on bone and renal complications associated with normocalcemic PHPT (52,53). Of note, neither study met the recommended diagnostic criteria for normocalcemic PHPT.

Bisphosphonate therapy is presumed to be as effective in patients with normocalcemic PHPT as in postmenopausal women or older men. One prospective open-label study evaluated the effects of oral alendronate in 30 postmenopausal women with normocalcemic PHPT (52). Patients were randomized to receive alendronate and cholecalciferol or cholecalciferol alone for 12 months. Lumbar spine, femoral neck and total hip bone mineral density (BMD) improved in the alendronate and cholecalciferol cohort by 4.7%, 2.6% and 4.0%, respectively, while BMD decreased significantly in the cholecalciferol-only group by –1.6%, –1.7% and –1.4%, respectively. Bisphosphonates therefore seem to be safe and effective for the treatment of osteoporosis in normocalcemic, as in hypercalcemic, PHPT. We would consider antiresorptive agents in patients with osteoporosis or with osteopenia and elevated fracture risk as calculated by the country-specific FRAX score. Of note, while densitometric changes in patients with PHPT treated with antiresorptive therapy appear similar to those in euparathyroid patients, there are no data to evaluate fracture risk reduction using antiresorptive therapy in patients with PHPT.

Another study evaluated the efficacy of cinacalcet in improving nephrolithiasis (53). Although the study consisted of only 6 patients with normocalcemic PHPT, cinacalcet reduced the number and size of urinary stones over a follow-up period of 10 months. Given the small sample size of this study, larger studies are needed to evaluate the impact of cinacalcet on patients with normocalcemic PHPT. Cinacalcet has been approved by the US Food and Drug administration (FDA) for the treatment of severe hypercalcemia in patients with PHPT who are unable to undergo parathyroidectomy (PTX), and by the European Medicines Agency (EMA) for the reduction of hypercalcemia in patients with PHPT, for whom PTX would be indicated on the basis of serum calcium levels (as defined by the relevant guidelines), but in whom surgery is not clinically appropriate or is contraindicated. Thus, in our opinion the use of cinacalcet in patients with normocalcemic PHPT patients is unreasonable.

#### Surgery

The Fourth International Workshop for the Management of Asymptomatic PHPT recommended PTX for normocalcemic PHPT if the patient developed a progression of the disease (such as worsening BMD) or new symptoms (i.e., fractures, kidney stones) in a very similar way to hypercalcemic patients (4). Similarly, the guidelines of the American Association of Endocrine Surgeons recommended surgery in symptomatic patients, without differentiating between hypercalcemic PHPT and normocalcemic PHPT (54,55). A recent expert consensus of the European Society of Endocrinology stated that surgical intervention should be considered only after experienced endocrine review; in this case, only if there are compelling indications and a surgical target (5). In our opinion, it is also important that discussion regarding surgery be individualized, and patient expectations also be comprehensively explored prior to surgery. We would consider surgery in patients with normocalcemic PHPT if the diagnosis has been clearly established with a long-standing history of elevated PTH values, symptoms, and a clear target on localization studies and/or referral to an experienced surgeon.

Preoperative localization studies are critical to the surgical management of PHPT. Precise preoperative localization may allow for a minimally invasive surgery with shorter operative time and decreased postoperative morbidity. Neck ultrasonography and ^99m^Tc-sestamibi scintigraphy are generally the first-line imaging tests to localize the hyperfunctioning gland(s) (56).

There are few investigations into imaging studies in patients with normocalcemic PHPT, and the data are unclear because most series do not fulfill the strict diagnostic criteria for normocalcemic PHPT (34,35,57–60). Moreover, most studies have selection bias given that they are mostly surgical case series. These studies are summarized in Table 3.

**Table 3:** Summary of imaging studies in cohorts of patients with normocalcemic primary hyperparathyroidism

Study	Population (n)	Detection rate (%)				Diagnostic criteria
		US	^99m^Tc-planar/SPECT-CT scintigraphy	4D-CT	18-F-Choline PET-CT	
Musumeci *et al.* (57)	32	75	-/59	-	-	Elevated PTH concentration with normal albumin-adjusted total serum calcium and normal ionized calcium; exclusion of renal failure (GFR < 60 mL/min), vitamin D deficiency (25-hydroxyvitamin D < 30 ng/mL), medication effect, hypercalciuria, gastrointestinal and malabsorptive diseases
Bossert *et al.* (58)	7	57	0/-	-	71	Elevated PTH concentration with normal albumin-adjusted total serum calcium and normal ionized calcium
Gómez-Ramírez *et al.* (59)	16	31.2	56/-	-	-	Elevated PTH concentration with normal albumin-adjusted total serum calcium and normal ionized calcium on repeat measure; exclusion of renal failure (GFR < 60 mL/min), vitamin D deficiency (25-hydroxyvitamin D < 30 ng/mL), medication effect, gastrointestinal and malabsorptive diseases
Cunha-Bezerra *et al.* (60)	8	2	11/-	56^1^	-	Elevated PTH concentration with normal albumin-adjusted total serum calcium; exclusion of renal failure (GFR < 60 mL/min), vitamin D deficiency (25-hydroxyvitamin D < 30 ng/mL), medication effect, hypercalciuria, gastrointestinal, liver and bone diseases
Šiprová *et al.* (35)	89	-	6/-	-	-	Elevated PTH concentration with normal albumin-adjusted total serum calcium and normal ionized calcium; exclusion of renal failure (GFR < 60 mL/min), vitamin D deficiency (25-hydroxyvitamin D < 20 ng/mL)^2^, medication effect
Wade *et al.* (34)	58^3^	50	40/-	-	-	Elevated PTH concentration with normal albumin-adjusted total serum calcium and normal ionized calcium

Abbreviations: US, neck ultrasonography; SPECT, single-photon emission computed tomography; 4D-CT, four-dimensional computed tomography.

^99m^Tc, Technetium (Tc)-sestamibi.

1 When considered the exact identification of the location (quadrant) the rate was 44.4.

2 Patients with 25-hydroxyvitamin D [25(OH)D] < 20 ng/mL were treated with vitamin D per os and serum 25(OH)D was examined again after 3 months. Only those patients in whom serum PTH remain increased after vitamin D substitution were included in the study.

3 The entire cohort of NPHPT was 89 but only in 58 was available ionized serum calcium.

Patients with normocalcemic PHPT generally have less positive localization studies compared to patients with classic hypercalcemic disease (35,60). The lower efficacy of localization studies is based on the higher prevalence of multiglandular disease and presence of smaller adenomas, a finding shared by most series (Table 3). The positivity rate of neck ultrasound ranges from 2 to 75% whereas that of scintigraphy from 0 to 56% depending on the population studied and the criteria used for exclusion of secondary causes of hyperparathyroidism. Of note, the positivity rate of single-photon emission computerized tomography (SPECT-CT) reported in a single study was 75% (57). Indeed, SPECT-CT, a combination of SPECT and CT acquisitions, is superior to each technique alone, particularly true in patients with ectopic glands, prior PTX, and coexistence of thyroid disease (56). Four-dimensional CT (4D-CT) has recently emerged as a promising technique for preoperative localization of parathyroid lesions in patients with PHPT (61–63). In one study, the sensitivity for identifying the parathyroid lesion, according to presentation of PHPT (hypercalcemic or normocalcemic), was found to be best with 4D-CT (56% positivity in normocalcemic vs. 75% in hypercalcemic) followed by ultrasound (22% vs. 58%), and scintigraphy (11% vs. 75%) (60). PET/CT with 18F-Fluorocholine (18F-FCH) has been shown to improve the detection of pathologic parathyroid glands in hypercalcemic PHPT. The diagnostic performance of 18F-FCH-PET/CT was evaluated in a small series of patients with normocalcemic PHPT (58). The detection rate 18F-FCH-PET/CT was of 69% vs. 61% for neck ultrasound, and a complete failure of scintigraphy to detect any hyperfunctioning parathyroid gland (58).

Normocalcemic PHPT has been reported to have a higher prevalence of multiglandular disease, ranging from 0 to 43% (Table 4) (35,59,64–70). Of note, multiglandular disease may decrease the success of preoperative localization and has implications for the technical difficulty of the operation by requiring bilateral cervical exploration (34,65,68,71). Several authors have recommended using intraoperative PTH (ioPTH) in this context to guide appropriate resection considering that its decline might take longer than that observed during PTX for classic PHPT (72). If more than one preoperative imaging technique provides concordant evidence for single-gland disease, then a focused exploration with ioPTH can be utilized. However, if ioPTH values indicate that single gland excision is not consistent with biochemical cure the surgeon should convert from a focused approach to four-gland exploration. An increased risk of conversion from a targeted approach to bilateral neck exploration has been reported in patients with normocalcemic compared to hypercalcemic PHPT (13% vs. 4%; p < 0.001, respectively) (68).

**Table 4:** Summary of the studies investigating the rate of multiglandular disease at surgery and the effect of parathyroidectomy on bone mineral density, nephrolithiasis and quality of life

Author	N	Design	Diagnostic criteria	Multiglandular disease (%)	Effect on		
					BMD	Nephrolithiasis	QoL
Gómez-Ramírez *et al.* (59)	16	Prospective	Elevated PTH concentration with normal albumin-adjusted total serum calcium and normal ionized calcium; exclusion of renal failure (GFR < 60 mL/min), vitamin D deficiency (25-hydroxyvitamin D < 30 ng/mL), medication effect, gastrointestinal diseases	7	lumbar spine (+33%) femoral neck (+20%); 1/3 distal radius (+15%	-	-
Sho *et al.* (64)^a^	71	Retrospective	Elevated PTH concentration with normal albumin-adjusted total serum calcium, ionized calcium in 59, 10/59 elevated; exclusion of renal failure (GFR < 60 mL/min), vitamin D deficiency (25-hydroxyvitamin D < 20 ng/mL)	-	+2.6 in the entire cohort; +5.6% in pts with normalized PTH, no significant change in patients with persistently elevated PTH	-	-
Pandian *et al.* (65)	733	Retrospective	Elevated PTH concentration with normal total serum calcium.	43	-	-	-
Lee *et al.* (66)			Elevated PTH concentration with normal albumin-adjusted total serum calcium	33	No change at any skeletal site (lumbar, femur and distal radius)	-	-
Traini *et al.* (67)^b^	154	Retrospective	Elevated PTH concentration with normal albumin-adjusted total serum calcium; exclusion of renal failure (GFR < 60 mL/min), vitamin D deficiency (25-hydroxyvitamin D < 20 ng/mL), medication effect, gastrointestinal diseases	13	Improvement in 5/12	No more evidence of kidney stones was observed in 4/10 patients, persistence in one and stability in 5	-
Trinh *et al.* (68)	119	Retrospective	Normal-range preoperative ionized calcium with elevated PTH levels	12	-	-	-
Bannani *et al.* (69)	32	Prospective	Serum calcium level at the upper limit of normal with an inappropriately high level of PTH; serum creatinine level over 160 μmol/l, patients taking lithium or thiazide diuretics, and those with other co-morbidities that could cause some of the symptoms evaluated or affect QoL.	0	-	-	Significant improvement in physical component summary score from the SF-36 questionnaire No significant improvement in mental component summary score At 1-year post-parathyroidectomy, significant improvement in two non-specific symptoms
Šiprová *et al.* (35)^c^	187	Prospective	Elevated PTH concentration with normal albumin-adjusted total serum calcium and normal ionized calcium; exclusion of renal failure (GFR < 60 mL/min), vitamin D deficiency (25-hydroxyvitamin D < 20 ng/mL), medication effect	0	-	-	-
Koumakis *et al.* (70)	39	Longitudinal	Elevated PTH concentration with normal albumin-corrected serum calcium, including 16 patients with normal ionized serum calcium; exclusion of renal failure (GFR < 40 mL/min), vitamin D deficiency (25-hydroxyvitamin D < 20 ng/mL), medication effect, gastrointestinal diseases	28.2	In the whole series, lumbar spine (+2.3%) and femoral neck (+1.9%); no change distal radius. In those with normal ionized serum calcium, femoral neck (+4.1%) and no change at the spine and distal radius	-	-

Abbreviations: BMD, bone mineral density; QoL, quality of life.

a Lowest DXA T-score (spine, femoral neck, or hip) preoperatively was chosen as the site where the pre- and postoperative.

b Post-operative bone mineral density was only available in 12 patients (not specified the site) and kidney ultrasound in 10 patients.

c Surgery was performed in 5 persistently normocalcemic patients who also had nodular goiter, and they were indicated for thyroidectomy.

Few data are available on outcomes of parathyroid surgery in normocalcemic PHPT (Table 4). These studies have shown an improvement in bone mineral density, nephrolithiasis, cardiovascular risk factors and quality of life. Of note, few studies fulfill the diagnostic criteria of normocalcemic PHPT.

Successful PTX has been reported to be followed at 1 year by a significant individual BMD gain in nearly half of normocalcemic PHPT patients with osteoporosis (Table 4) (59,64,67,70).

Only one study evaluated the effect of PTX on nephrolithiasis. The study included only 10 patients, with no further evidence of kidney stones present in 4/10 patients, persistence in one and stability in five (Table 4) (67).

One study showed that blood pressure, serum total cholesterol, and homeostatic model assessment-insulin resistance (HOMA-IR) improved after PTX (73). Conversely, neither glycated hemoglobin levels nor the homeostatic model assessment-beta cell function (HOMA-B) index changed in patients with normocalcemic PHPT and prediabetes undergoing parathyroid surgery or conservative follow-up for up to 8 months, indicating a minor impact of parathyroid surgery on these outcomes (74). Of note, the latest guidelines on the management of asymptomatic PHPT (4) did not recommend parathyroid surgery to improve overall cardiovascular risk either for mild traditional hypercalcemic or normocalcemic PHPT because of the lack of evidence and contradictory findings.

The question of whether PTX can improve quality of life (QoL) is still uncertain in patients with classic PHPT and only one study evaluated this question in patients with normocalcemic PHPT (Table 4) (69). QoL was assessed by self-administered Short Form-36 v.2 Questionnaire. Patients with normocalcemic PHPT had improvement in some QoL domains after PTX. There was an improvement in anxiety at 3 months, while only thirst was still improved at 6 months, and thirst and fatigue at 12 months.

In conclusion, the literature on normocalcemic PHPT is based mostly on larger studies of population-based cohorts and smaller studies from referral centers. Few studies have rigorously followed the definition of normocalcemic PHPT given by the latest guidelines, which require that serum total or ionized calcium should be normal on repeated measurements over time. The lack of rigorous diagnostic criteria and selection bias may explain the heterogeneity of reported rates of bone and renal complications in relation to consistently mild laboratory alterations. Moreover, there are questions regarding the significance of normocalcemic PHPT when it is just a biochemical signature in a patient without bone or kidney complications. Finally, its natural history remains still to be elucidated because a proportion of patients diagnosed with normocalcemic PHPT may spontaneously revert to having normal PTH levels. With all these concerns, caution should be used in recommending surgery for normocalcemic PHPT. Surgery should be considered only in the setting of an expert multidisciplinary approach and only if there are compelling indications and a surgical target.
